# International Gender and Alcohol Research: Recent Findings and Future Directions

**Published:** 2002

**Authors:** Sharon C. Wilsnack, Richard W. Wilsnack

**Affiliations:** Sharon C. Wilsnack, Ph.D., is the Chester Fritz Distinguished Professor, and Richard W. Wilsnack, Ph.D., is a professor, both in the Department of Neuroscience at the University of North Dakota School of Medicine and Health Sciences, Grand Forks, North Dakota

As international travel, migration, and mass media increase cross-national influences on alcohol use and alcohol-related problems, better understanding of the sociocultural influences on women’s and men’s drinking will become increasingly valuable for improving national and international efforts to reduce problems caused by alcohol consumption. This article summarizes international collaborative research conducted during the past decade on gender-related influences on alcohol use, identifies future directions for research in this area, and suggests possible implications of this research for the development of more gender-sensitive national and international alcohol policy.

## Precursors of International Gender and Alcohol Research

International comparative research on alcohol use (comparing two or more countries or cultures) developed in the 1970s, initially to examine alcohol control policies (Bruun et al. 1975) and community responses to alcohol use (Rootman and Hawks 1987). Subsequent research included an international study that compared Alcoholics Anonymous in different countries (Mäkelä et al. 1996), a study that followed up the work of Bruun and colleagues comparing alcohol policies in various countries (Edwards et al. 1994), a 9-country study that used a variety of qualitative and quantitative methods to evaluate the cross-cultural applicability of diagnostic criteria and assessment instruments for substance use disorders (Room et al. 1996), a World Health Organization (WHO) project to develop international screening methods for harmful alcohol use (Saunders et al. 1993), and meta-analyses of data from more than 38 longitudinal alcohol surveys in 15 countries (Fillmore et al. 1991, 1997).

Paralleling the development of international research on drinking behavior, gender’s influence on drinking patterns and problems has also been the subject of increased attention as a result of research in the United States and Canada (e.g., the work of Ferrence, Fillmore, Graham, Nadeau, and S. and R. Wilsnack; see Wilsnack and Beckman 1984) as well as in Europe (including work by Ahlström, Haavio-Mannila, and Holmila in Finland; Kubicka in the Czech Republic; Neve in the Netherlands; and Spak in Sweden; see Haavio-Mannila 1989; Kubicka et al. 1995; Neve et al. 1996). Pioneering meta-analyses of international longitudinal surveys (Fillmore et al. 1997) found that, consistently across cultures, men drank more than women and that marriage and aging reduced both women’s and men’s drinking. Depression predicted subsequent increases in drinking among women but not among men. Such quantitative research has been complemented by a growing number of ethnographic studies on cultural patterns in the differences between men’s and women’s drinking (e.g., Gefou-Madianou 1992; McDonald 1994).

International research on gender and alcohol use has increased substantially in recent years. The *Current Contents* database of publications in scientific journals in the years 2000 and 2001 lists more than 100 studies conducted outside the United States that analyzed gender-differentiated data on alcohol use or alcohol-related problems. Approximately 20 of these studies compared 3 or more ethnic groups or countries. A recent monograph contains published and unpublished reports of gender-specific drinking patterns and trends in Latin America and the Caribbean, the region with the world’s highest percentage of deaths attributable to alcohol (Pyne et al. 2002). This article cannot provide a comprehensive review of all the international alcohol research involving gender comparisons. Instead, it focuses on one systematic research effort—by the International Research Group on Gender and Alcohol (IRGGA)—to coordinate research in this area and to design new methods and measures that will allow future research on gender and alcohol use to be more comparable across countries.

## International Research Group on Gender and Alcohol

The evolution of international comparative alcohol research and gender-focused alcohol research converged in the organization of IRGGA in 1993. IRGGA’s membership now includes more than 100 alcohol researchers from 35 countries.

The first task facing IRGGA was to develop standard reporting units of alcohol consumption that could be used for comparing most or all of the survey data sets compiled by IRGGA members. The process of developing standard units revealed enormous variation in how alcohol use and alcohol problems were measured in different countries and raised interest in planning a future international study in which all countries would use comparable measures to collect new data on women’s and men’s drinking behavior.

### Methodological Issues: Gender Adjustments for Alcohol Consumption?

One of the first methodological questions confronting IRGGA was whether the common measures of alcohol consumption should use a “gender correction factor” that took into account the fact that women reach higher blood alcohol levels than men when consuming equivalent weight-adjusted amounts of alcohol. Hypothetically, lack of gender adjustment could result in underestimating women’s hazardous alcohol consumption and could lead women to underestimate the behavioral and health effects of their seemingly moderate levels of consumption. Based on a review and analysis of the relevant research literature, an IRGGA work group concluded that universal gender adjustments of consumption levels are not presently justified because other aspects of drinking behavior may modify biological gender effects. Among these possible characteristics are gender differences in drinking styles and pace, the amount of food consumed while drinking, beverage choice, and drink sizes, as well as potential interactions of alcohol with other substances, including prescription medications (Graham et al. 1998). As a result of this review, subsequent IRGGA analyses have used the same measures of alcohol consumption for women and men.

### Gender and Alcohol Use in 10 Countries

Using the standard measures developed by IRGGA, researchers compared women’s and men’s drinking in 16 general population surveys from 10 countries: Australia, Canada, the Czech Republic, Estonia, Finland, Israel, the Netherlands, Russia, Sweden, and the United States (Wilsnack et al. 2000). In all countries, men were more likely to drink than women, and male drinkers consumed alcohol more frequently and in larger amounts, and were more likely to have alcohol-related problems than female drinkers. The consistency of this pattern across countries (and in other research literature) suggests that gender differences in drinking behavior may be biologically influenced. However, substantial variation in the *magnitude* of gender differences across countries suggests that the differences are also strongly influenced by sociocultural factors. For example, according to statistics on monthly frequency of drinking, in the Netherlands men drank 1.43 times as often as women, and in Israel men drank 3.24 times as often as women (Wilsnack et al. 2000). One hypothesis developed from these analyses proposes that relatively small biological differences in how alcohol affects women and men are magnified by cultural rules for how women and men should or should not drink. One reason why gender differences in drinking effects may be culturally magnified is that differences in drinking behavior may be a useful way to symbolize more general differences in gender roles and to make gender role differences more conspicuous. Thus, many societies with major differences in men’s and women’s roles have also largely forbidden women (but not men) to drink (McDonald 1994; Medina-Mora 1994).

### Alcohol Consumption and Consequences in Five Countries

Another collaborative study examined relationships between women’s drinking and women’s alcohol-related problems in five countries (Australia, the Czech Republic, the Netherlands, Sweden, and the United States) (Vogeltanz-Holm et al. in press). In all five countries the amount of alcohol consumed per day was a better predictor of alcohol dependence symptoms than was frequency of drinking. However, in all countries except the United States, women’s frequency of drinking was slightly better than women’s drinking quantity per occasion for predicting whether their drinking would be criticized. Contrary to the study’s hypothesis, the relationship between alcohol consumption and social criticism of drinking was not consistently stronger in countries where women’s drinking is less accepted or tolerated. These findings suggest that a social deviance model (in which social reactions are more negative or punitive toward behavior that deviates to a greater degree from what other people expect) may not be a good explanation of cross-cultural variations in women’s drinking-related problems. Better indicators of women’s drinking norms are needed before definitive conclusions can be drawn.

### Qualitative Research in IRGGA

One topic of discussion throughout IRGGA’s history has been how quantitative and qualitative methods can be used together to improve the design of survey instruments and to better understand the psychological and social processes that underlie quantitative research findings. Qualitative research in IRGGA has included a study in which members conducted in-depth interviews with women and men in six countries about their reactions to gender-related images in alcohol beverage advertisements, and a workshop on the use of qualitative methods in international gender and alcohol research. Directors of country surveys for the group’s new GENACIS project (described below) have been encouraged to incorporate qualitative substudies to aid in interpreting their quantitative survey findings.

## European Union “Women and Alcohol in Europe” Project

In 1996 a subset of IRGGA members from European Union (EU) countries conducted a comparative study of women’s and men’s drinking patterns, and their acute and chronic consequences, in nine European countries: Finland, France, Germany, Great Britain (Scotland), Italy, the Netherlands, Sweden, Switzerland, and the Czech Republic. The EU group investigated whether European women’s and men’s drinking patterns were becoming more similar (the “convergence” hypothesis). The researchers concluded that any such convergence has been small, inconsistent, and statistically uncertain in most European countries, with the exception of Finland (where until recently women were exceptionally likely to be abstainers or very infrequent drinkers). In none of the European countries studied did women’s drinking frequencies or quantities exceed those of men (Bloomfield et al. 2001).

The researchers also considered whether recent changes in women’s roles, in particular increased education and more paid employment outside the home, had resulted in increased drinking by women (the “emancipation” hypothesis). The data showed that women with higher levels of education were more likely to drink at least occasionally (i.e., not to abstain completely) (Allamani et al. 2000). However, more complex analyses found that paid employment, by itself or in combination with other roles such as marriage and parenthood, did not consistently raise the likelihood that women would drink *heavily* (12 or more drinks per week) (Gmel et al. 2000).

The EU study also addressed the question of how survey measures of drinking behavior could be made more gender-sensitive. The investigators concluded that oversimplified questions (e.g., questions that are not beverage-specific, do not specify drink sizes, and do not ask about atypical drinking occasions) should be avoided because they underestimate women’s drinking more than men’s, leading to overestimation of gender differences. They also recommended the evaluation of cultural effects on the reliability and validity of drinking measures (Knibbe and Bloomfield 2001).

## Current International Gender and Alcohol Research: The GENACIS Project

International research using secondary analyses of existing data sets has been hampered by differences in how drinking is measured (e.g., drinks, bottles, glasses, milliliters) and by differences in how drinking consequences are measured (with different countries emphasizing different types of consequences). These differences make it difficult to compare drinking behavior and its consequences across countries.

Drawing on their experience in collaborative research, IRGGA members have designed a new international study, known as GENACIS (Gender, Alcohol, and Culture: An International Study). GENACIS is obtaining new survey data using a standardized set of questions. At the time of this writing, 30 countries have completed, are conducting, or plan to conduct surveys as part of GENACIS (see table). Some surveys are being conducted specifically for GENACIS, and some surveys designed for other purposes (e.g., national health or alcohol use surveys) will include sets of GENACIS questions. Funding from the European Union is supporting coordination and centralized analysis of data from GENACIS surveys in 13 EU member states and associated states. The WHO has provided funding for GENACIS surveys in several countries, presently including Argentina, Costa Rica, India, Kazakhstan, Nigeria, Sri Lanka, and Uganda. Additional funding is provided by the National Institute on Alcohol Abuse and Alcoholism to support worldwide coordination of GENACIS and to assist countries not eligible for funding from the EU or WHO.

The GENACIS questionnaire developed by IRGGA contains questions about drinking behavior, drinking contexts (where, when, and with whom a person drinks), and drinking consequences (as perceived both by the respondent and by other people), and includes alcohol dependence questions from the Alcohol Use Disorders Identification Test (AUDIT) (Saunders et al. 1993). The questionnaire attempts to assess consumption not only of legal commercial alcoholic beverages but also of illicit or unrecorded alcoholic beverages (e.g., home brew, moonshine, and smuggled alcohol), which in some developing countries constitute most of the alcohol consumed (Green 1999; Pyne et al. 2002). In addition to measures of alcohol consumption, the GENACIS questionnaire asks about various life domains that may affect or be affected by alcohol use, including social networks and social support, social roles (employment, marriage, parenthood), intimate relationships and sexuality, experiences of violence and victimization, physical and emotional health, use of prescribed and illicit drugs, and excessive or compulsive behavior such as eating or gambling. GENACIS will also evaluate the effects of *societal-level* characteristics such as (1) gender inequality (using indicators such as female and male education, employment, income, and political participation), and (2) differences between “wet” drinking cultures (with high prevalence of alcohol use and more permissive drinking norms) and “dry” drinking cultures (with higher rates of abstinence from alcohol and more restrictive drinking norms) (Allamani et al. 2000).

Centralized analyses of GENACIS data are designed through collaboration of survey directors and other project partners interested in specific research questions. Data analysis is expected to continue for several years, examining cross-national similarities and differences in women’s and men’s drinking patterns and drinking-related problems, and in the individual- and societal-level predictors of gender-specific drinking patterns and drinking problems. The unique GENACIS data will be archived for future use to study such things as global time trends in women’s and men’s alcohol use, its predictors, and its consequences.

## Possible Implications of International Gender and Alcohol Research for International Alcohol Policy

Research examining alcohol and gender issues worldwide, as described above, reveals several issues that have important implications for international alcohol policy.

### Importance of Culture

Recent international research on gender and alcohol has clearly demonstrated that programs and policies that try to be gender-sensitive cannot ignore cultural influences. Large cross-national variation in gender differences in drinking behavior indicates that biological factors alone cannot account for differences in how women and men drink. To be gender-sensitive, education, prevention and treatment programs, and alcohol policies must take into account both biological differences in alcohol effects *and* culturally defined gender roles that specify expected and tolerated drinking behavior for women and men. For example, prevention strategies targeting social norms that promote high-risk drinking behaviors among males may be particularly appropriate and needed in countries with highly gender-differentiated drinking behavior (Pyne et al. 2002).

**Table:** GENACIS Survey

Completed	
Argentina	Japan
Australia	Kazakhstan
Austria	Mexico
Brazil	Netherlands
Czech Republic	Nigeria
Finland	Norway
France	Spain
Germany	Sri Lanka
Hungary	Sweden
Iceland	Switzerland
Israel	United Kingdom
Italy	United States

**In Progress**	
Canada	
Costa Rica	
India	
Uganda	

**Planned**	
Denmark	
Russia	

**Potential**	
Baltic Countries	
China	
Moldova	
New Zealand	
Ukraine	

NOTE: An international study on gender and alcohol known as GENACIS (Gender, Alcohol, and Culture: An International Study) is currently under way. Thirty countries have completed, are conducting, or plan to conduct surveys as part of GENACIS.

### Definitions of Hazardous Drinking

Publicized guidelines for high- and low-risk drinking should be better informed by research on gender and alcohol. Most countries with such standards recommend lower limits of alcohol consumption for women than for men, but the levels of consumption defined as “safe” vary considerably across countries. For example, Australia defines low-risk consumption as no more than 28 drinks per week for men or 14 drinks per week for women (National Health and Medical Research Council 2001), whereas U.S. standards of low-risk drinking are no more than 14 drinks per week for men and 7 drinks per week for women^1^ (National Institute on Alcohol Abuse and Alcoholism 1995). Guidelines for safe as opposed to hazardous drinking should be based on valid data from scientifically representative samples of women and men and should not be adopted based on subjective judgment or imitation of other countries. Ideally, gender-specific warnings about alcohol use should be tailored to the *type* of alcohol-related problem to be reduced. For example, educating women about drinking during pregnancy would use a different definition of “risk drinking” than that used in educating men about alcohol-related domestic violence. Consumption levels with relatively low risks for *acute* alcohol-related health problems (e.g., having three or four drinks at a social celebration) might be much too high for *chronic* daily alcohol consumption. Demographic and cultural characteristics may also affect “safe” levels of drinking: consumption levels that are risky for elderly retirees in Florida may not be as risky for young businesswomen in Oslo or Prague.

### Stigmatization of Women’s Drinking

Double standards of drinking that judge excessive (or any) alcohol use more harshly for women than for men have been reported in many cultures throughout history (e.g., Blume 1997). This stigmatization may be greater in highly gender-differentiated societies in which women are allowed little if any access to alcohol (Ikuesan 1994; Medina-Mora 1994; Mphi 1994). Greater stigmatization of women’s drinking, resulting in greater guilt and shame for women who do drink and for their families, has at least two important implications for health policy. First, women with drinking problems in these societies may be reluctant to seek treatment or may be prevented by ashamed family members from seeking treatment. Second, where women’s drinking problems are stigmatized, the prevalence of such problems may be seriously underestimated, and policymakers may not know how much harm alcohol is causing women.

### Effects of Changing Gender Roles

A final implication of international gender and alcohol research for alcohol policy is that changes in women’s and men’s roles are likely to be accompanied by changes in women’s and men’s drinking behavior. When women improve their education, employment, and status, they are likely also to have more opportunities to drink. However, this does *not* imply that women with higher levels of education and higher status jobs are in greater danger of becoming *problem* drinkers. It does imply that where women gain increasing education, income, and status, prevention of problem drinking will have to be achieved primarily at the individual level, because social environments will no longer inhibit all drinking by all women.

Finally, if women’s education and employment improve, what will happen to *men’s* drinking? If improvements in women’s roles threaten the self-worth of some men, or if women have better employment opportunities than men do, will changes in women’s roles increase risks of problem drinking among men? This question may deserve greater attention from alcohol problem prevention specialists as gender roles become defined more by global standards and less by local traditions.

## Conclusions

As the world becomes “smaller,” it will become increasingly important and valuable to compare the patterns, predictors, and consequences of women’s and men’s alcohol use across varying cultural contexts. In addition to improving the culture- and gender-sensitivity of measures of alcohol use and alcohol problems, cross-national research can help to explicate the complex interactions of biology with other individual-level and societal-level variables that influence the drinking behavior of women and men. Such research can ultimately inform the design of better-targeted and more effective prevention and intervention efforts for women and men worldwide.

## Footnotes

1 The difference between Australian and U.S. guidelines is reduced somewhat by differences in drink sizes. A standard drink in Australia is defined as 10 grams of ethanol (absolute alcohol) (National Health and Medical Research Council 2001), and a U.S. standard drink is defined as 12 to 14 grams (Zernig et al. 2000). The resulting definition of low-risk drinking for women is no more than 140 grams in Australia and no more than 84 to 98 grams in the United States.

## References

[b1-245-250] Allamani A, Voller F, Kubicka L, Bloomfield K (2000). Drinking cultures and the position of women in nine European countries. Substance Abuse.

[b2-245-250] Bloomfield K, Gmel G, Neve R, Mustonen H (2001). Investigating gender convergence in alcohol consumption in Finland, Germany, the Netherlands and Switzerland: A repeated survey analysis. Substance Abuse.

[b3-245-250] Blume SB, Wilsnack RW, Wilsnack SC (1997). Women and alcohol: Issues in social policy. Gender and Alcohol: Individual and Social Perspectives.

[b4-245-250] Bruun K, Edwards G, Lumio M (1975). Alcohol Control Policies in Public Health Perspective.

[b5-245-250] Edwards G, Anderson P, Babor T (1994). Alcohol Policy and the Public Good.

[b6-245-250] Fillmore KM, Hartka E, Johnstone BM (1991). A meta-analysis of life course variation in drinking. British Journal of Addiction.

[b7-245-250] Fillmore KM, Golding JM, Leino EV, Wilsnack RW, Wilsnack SC (1997). Patterns and trends in women’s and men’s drinking. Gender and Alcohol: Individual and Social Perspectives.

[b8-245-250] Gefou-Madianou D (1992). Alcohol, Gender, and Culture.

[b9-245-250] Gmel G, Bloomfield K, Alhström S (2000). Women’s roles and women’s drinking: A comparative study in four European countries. Substance Abuse.

[b10-245-250] Graham K, Wilsnack R, Dawson D, Vogeltanz N (1998). Should alcohol measures be adjusted for gender differences?. Addiction.

[b11-245-250] Green M (1999). Trading on inequality: Gender and the drinks trade in southern Tanzania. Africa.

[b12-245-250] Haavio-Mannila E (1989). Women, Alcohol, and Drugs in the Nordic Countries.

[b13-245-250] Ikuesan BA (1994). Drinking problems and the position of women in Nigeria. Addiction.

[b14-245-250] Knibbe R, Bloomfield K (2001). Alcohol consumption estimates in surveys in Europe: Comparability and sensitivity for gender differences. Substance Abuse.

[b15-245-250] Kubicka L, Csemy L, Kozeny J (1995). Prague women’s drinking before and after the “Velvet Revolution” of 1989: A longitudinal study. Addiction.

[b16-245-250] Mäkelä K, Arminen I, Bloomfield K (1996). Alcoholics Anonymous as a Mutual-Help Movement: A Study in Eight Societies.

[b17-245-250] McDonald M (1994). Gender, Drink, and Drugs.

[b18-245-250] Medina-Mora E (1994). Drinking and the oppression of women: The Mexican experience. Addiction.

[b19-245-250] Mphi M (1994). Female alcoholism problems in Lesotho. Addiction.

[b20-245-250] National Health and Medical Research Council (2001). Australian Alcohol Guidelines: Health Risks and Benefits.

[b21-245-250] National Institute on Alcohol Abuse and Alcoholism (NIAAA) (1995). The Physician’s Guide to Helping Patients with Alcohol Problems.

[b22-245-250] Neve R, Drop M, Lemmens P, Swinkels H (1996). Gender differences in drinking behavior in the Netherlands: Convergence or stability?. Addiction.

[b23-245-250] Pyne HH, Claeson M, Correia M (2002). Gender Dimensions of Alcohol Consumption and Alcohol-Related Problems in Latin America and the Caribbean.

[b24-245-250] Room R, Janca A, Bennett LA (1996). WHO cross-cultural applicability research on diagnosis and assessment of substance use disorders: An overview of methods and selected results. Addiction.

[b25-245-250] Rootman I, Hawks D (1987). Some reflections on the WHO project on community response to alcohol-related problems. British Journal of Addiction.

[b26-245-250] Saunders J, Aasland O, Babor T (1993). Development of the Alcohol Use Disorders Identification Test (AUDIT): WHO Collaborative Project on Early Detection of Persons with Harmful Alcohol Consumption—II. Addiction.

[b27-245-250] Vogeltanz-Holm ND, Neve RJM, Greenfield TK A cross-cultural analysis of women’s drinking and drinking-related problems in five countries: Findings from the International Research Group on Gender and Alcohol. Addiction Theory and Research.

[b28-245-250] Wilsnack SC, Beckman LJ (1984). Alcohol Problems in Women: Antecedents, Consequences, and Intervention.

[b29-245-250] Wilsnack RW, Vogeltanz ND, Wilsnack SC (2000). Gender differences in alcohol consumption and adverse drinking consequences: Cross-cultural patterns. Addiction.

[b30-245-250] Zernig G, Saria A, Kurz M, O’Malley SS, Zernig G, Saria A, Kurz M, O’Malley SS (2000). Definitions of a “standard drink”. Handbook of Alcoholism.

